# Prediction and Fitting of Nonlinear Dynamic Grip Force of the Human Upper Limb Based on Surface Electromyographic Signals

**DOI:** 10.3390/s25010013

**Published:** 2024-12-24

**Authors:** Zixiang Cai, Mengyao Qu, Mingyang Han, Zhijing Wu, Tong Wu, Mengtong Liu, Hailong Yu

**Affiliations:** 1 School of Chemistry and Chemical Engineering, Shanghai Jiao Tong University, Shanghai 200240, China; 2 School of Electronic Information and Electrical Engineering, Shanghai Jiao Tong University, Shanghai 200240, China; 3 School of Biomedical Engineering, Shanghai Jiao Tong University, Shanghai 200240, China

**Keywords:** surface electromyographic signals, nonlinear dynamic grip force, NARX neural network, grip force prediction

## Abstract

This study aimed to predict and fit the nonlinear dynamic grip force of the human upper limb using surface electromyographic (sEMG) signals. The research employed a time-series-based neural network, NARX, to establish a mapping relationship between the electromyographic signals of the forearm muscle groups and dynamic grip force. Three-channel electromyographic signal acquisition equipment and a grip force sensor were used to record muscle signals and grip force data of the subjects under specific dynamic force conditions. After preprocessing the data, including outlier removal, wavelet denoising, and baseline drift correction, the NARX model was used for fitting analysis. The model compares two different training strategies: regularized stochastic gradient descent (BRSGD) and conjugate gradient (CG). The results show that the CG greatly shortened the training time, and performance did not decline. NARX demonstrated good accuracy and stability in dynamic grip force prediction, with the model with 10 layers and 20 time delays performing the best. The results demonstrate that the proposed method has potential practical significance for force control applications in smart prosthetics and virtual reality.

## 1. Introduction

Surface electromyographic (sEMG) signals generated by muscle contractions are widely used for the recognition of human movement intention. These signals can be recorded by sensors and used to identify different actions or gestures. Due to their ease of collection, non-invasiveness, richness, and naturalness, sEMG signals are highly suitable as input sources for various human–computer interaction control systems. In recent years, this area has garnered widespread academic attention, with ongoing research and development leading to significant advances and preliminary applications in fields such as rehabilitation medicine, prosthetics and exoskeleton control, human–machine interaction (HMI), and health monitoring and prevention [1,2,3,4].

However, the use of sEMG signals to predict muscle force still needs to be further investigated. This is because the prediction of muscle force is indispensable if a safe and smooth HMI system is to be achieved. For instance, the development of smart rehabilitation devices is increasingly driven by the need for precise force control. Most can only recognize specific actions and do not control the force applied by the prosthetic hand. In addition to smart prosthetics, many human–computer interaction devices using sEMG signals as a control input focus primarily on action recognition, often overlooking force control. During gripping, human hands not only perform specific actions but also apply varying forces, such as grip strength or pinch force. Simple action recognition alone is insufficient for achieving natural and functional prosthetic control. This can affect user experience and even safety. For example, in prosthetic hands, applying excessive force may deform or damage objects, while insufficient force might cause the prosthetic hand to drop objects. Thus, precise force control using sEMG signals is crucial [3].

Establishing a mapping relationship between dynamic grip force and electromyographic signals holds significant long-term potential. Dexterous hands are essential tools in daily life and work, playing a crucial role in various activities. Electromyographically controlled prosthetic hands are a key focus in current commercial development [5,6,7]. For prosthetics to effectively replace human hands, they must simulate the posture and force application of real hands. In many real-world scenarios, continuous dynamic grip force control is required, which necessitates a mapping between EMG signals and dynamically changing grip force. Integrating this mapping into smart prosthetics would enable them to mimic human-like dynamic grip control.

Many researchers are dedicated to developing methods for estimating muscle force based on sEMG signals. Kim et al. proposed a method for estimating fixed static forces based on sEMG signals [8]. This method estimates a fixed grip force value in scenarios with a constant force output. Wu et al. investigated the relationship between electromyographic signals and muscle force, demonstrating a good linear correlation between the two and confirming that force changes can be inferred from electromyographic signals [9]. In their work, for force decoding, the authors chose the most direct and simplest method of linear grip force control, analyzing the rate of linear change in grip force per unit time based on electromyographic signals and establishing a mapping relationship between linear grip force and electromyographic signals. While these studies extensively explored instantaneous, static, and linear grip forces, there is still a lack of specific investigation into continuously varying dynamic grip forces. Al-Timemy et al. also noted that changes in force can result in a 60% reduction in the accuracy of the electromyographic control system [10]. Subsequently, they investigated the robustness control of hand prostheses under varying force levels and found that changes in force may have a greater impact on the robustness of prosthetic control [11]. In Ma et al.’s study, the BP neural network algorithm was employed to fit hand force based on sEMG signals. Their model reduced the MSE to approximately 0.01 using 16-channel sEMG signals [3]. However, their research was limited to pinch movements of the fingers. Considering that in real-life scenarios, people tend to use grasping actions involving the entire palm for handling heavy objects, their findings have limited relevance to practical application scenarios. Bardizbanian et al. conducted a detailed investigation into one-time EMG force models, where a new model is trained each time the electrodes are reapplied [12]. They used 16-channel electrodes to collect EMG signals, but the large data volume resulted in training times of at least 14 s, with most exceeding 30 s and even reaching 60 s. Clearly, such extended training times are not conducive to practical applications. Moreover, the study faced significant challenges in electrode placement due to the fixed equidistant arrangement of the 16 electrodes, which limited the selection of optimal locations. Additionally, the excessive number of electrodes poses a burden for users in real-world scenarios.

Therefore, one of the most important goals of force decoding from EMG signals is to estimate the required grip force in real life, in real time, based on sEMG signals, and to accurately reflect this force in prosthetics or other devices. However, the use of sEMG signals as a source of force control signals for smart prosthetics faces two problems. Firstly, it is necessary to utilize as few channels as possible for force analysis to meet the requirements of practical applications to reduce the computational load and training time during training. Second, dynamic force estimation rather than static calibration needs to be performed using sEMG signals. Due to the limitations of experimental requirements and algorithms, the above aspects need to have further research.

sEMG signals are generated by muscle contractions, with different muscle groups playing dominant roles depending on the type of movement and force applied. The strength of force in the forearm is closely related to the extent of muscle contraction. During gripping, the force exerted by different fingers varies, and each muscle has a specific function. Multiple muscles work together to complete a movement pattern. The forearm muscle distribution is complex, with different movement patterns potentially involving the same muscle groups or varying muscle groups for the same pattern. The closer a muscle is to the hand, the more its contractions are related to hand movement patterns [13]. Therefore, understanding the structure and distribution of forearm muscles is crucial for selecting target muscle groups for signal acquisition in experiments [10], leading to better models for analyzing movement conditions.

The forearm muscles, located around the radius and ulna, are divided into anterior and posterior groups, totaling over 20 muscles. Most of these are long muscles with long tendons; the muscle belly is proximal, while the tendons are distal, making the upper part of the forearm thicker and the lower part thinner. The anterior group mainly includes muscles for wrist flexion, finger flexion, and forearm pronation, located on the front and medial side of the forearm. The posterior group includes muscles for wrist extension, finger extension, and forearm supination, located on the back and lateral side of the forearm.

The aim of this paper is to find a suitable algorithm to establish the mapping relationship between the electromyographic signals of forearm muscle groups and dynamic grip force. And the experiment needed to be conducted in a dynamic force environment to ensure that the experimental conditions meet the requirements of practical applications. The experimental results verify the effectiveness of the proposed scheme.

## 2. Materials and Methods

### 2.1. Experiment Design for Subjects

In the signal acquisition experiment, we developed a system that can simultaneously collect sEMG and grip force signals. A three-channel sEMG signal acquisition device was used, which supports wireless data transmission. The sensors had a sampling frequency of 1000 Hz, and the collected signals were filtered between 10 and 1000 Hz. Additionally, the grip force was measured using an S-type grip force sensor with a sampling frequency of 10 Hz. In practical data usage, we performed downsampling on the preprocessed sEMG signals, reducing their sampling rate to 10 Hz to align with the sampling frequency of the force signals. While downsampling inevitably leads to the loss of signal details and a decrease in the fitting accuracy of the model’s output, we consider this operation acceptable if the results remain within an acceptable range. This is because downsampling significantly reduces the time required for model training and computational overhead [14].

**Figure 1:**
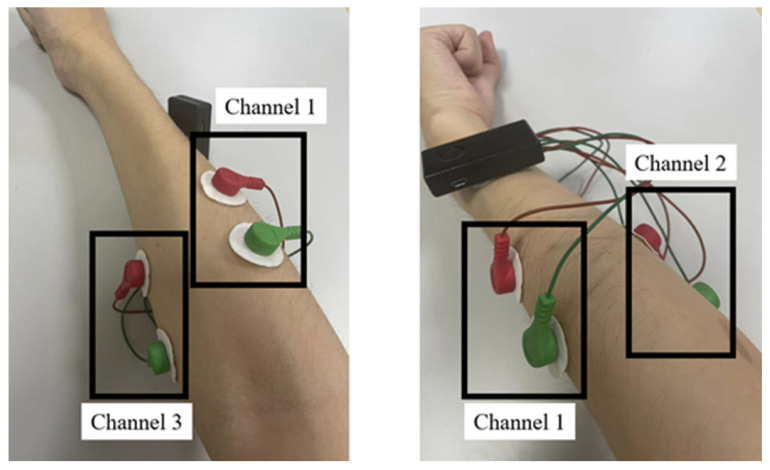
Electrode placement.

Hand and wrist movements depend on the coordinated action of multiple muscles, and different hand movements involve different muscle groups. To enhance the accuracy and representativeness of action recognition, this study selected three specific muscles for sEMG signal collection. These muscles are the brachioradialis (Channel 1), extensor carpi ulnaris (Channel 2), and flexor carpi ulnaris (Channel 3) (Figure 1).

The selection of the brachioradialis, ulnar wrist extensor, and ulnar wrist flexor muscles as the detection targets when performing EMG signal acquisition for continuous fist-clenching movements is based on the important role and specific physiological properties of these muscles in hand movements. First, from an anatomical point of view, the three muscles, brachioradialis, ulnar wrist extensor, and ulnar wrist flexor, are located in the superficial layer of the forearm and encircle the forearm in a circle [15,16]. This location advantage makes it easier for the electrodes to fit on the muscle surface when performing EMG signal acquisition, thus acquiring clear signals. In addition, since these muscles are in the surface layer, there is less interference during signal acquisition, which helps to improve the quality and reliability of EMG signals. Meanwhile, fist clenching not only involves finger flexion but also requires the concerted work of forearm muscles to accomplish fine hand control and force output [17]. The contraction of the brachioradialis muscle, as one of the major flexor muscles of the forearm, is crucial for the fist-clenching action [18], whereas the ulnar extensor carpi radialis muscle and ulnar flexor carpi ulnaris muscle play important roles in stabilizing the wrist joint and regulating the finger movements [19]. By detecting the activities of these muscles, the dynamic changes in muscle coordination during the fist-clenching process can be captured, providing rich data support for subsequent signal processing and applications.

Further, considering the application of myoelectric prosthesis, the designed EMG acquisition device can be more compactly wrapped around the forearm for easy wearing and use by the user due to the highly approximate position of these three muscles. This not only improves the comfort of the device but also enhances the stability and consistency of signal acquisition. Also, since amputations below the wrist usually do not damage the muscles located in the forearm, the user can still control the prosthesis through the flexor and extensor muscles of the forearm [20]. Therefore, the acquisition of EMG signals from these areas can be used for operational control of the prosthesis, helping the user to achieve a more natural hand function.

In this experiment, thirteen healthy participants aged 21–25 were selected for the study, including 10 males and 3 females, all of whom were right-handed. The participants had no history of neurological or musculoskeletal disorders and were provided with a written informed consent form. The experiment took place in a normal indoor environment, free from large equipment and severe noise interference. To collect accurate and effective EMG signals, the participants refrained from vigorous exercise before the experiment, kept their muscles naturally relaxed, and avoided muscle fatigue to prevent its impact on the EMG signals. The subjects were instructed to follow prompts from the experimenter. They first had three sets of hydrogel wet electrodes attached to the right forearm for measuring sEMG signals from the targeted muscles. The right hand was then positioned in a relaxed grip on the load cell sensor in preparation for detection. Under the experimenter’s direction, subjects were required to gradually increase their grip strength to firmly grasp the load cell sensor from a relaxed state. Each grip was held for 5 s, with a 5 s interval between each grip, and the process was repeated three times.

### 2.2. Data Preprocessing

#### 2.2.1. Outlier Removal and Filtering

The sEMG signals are closely related to neuromuscular physiological activities and are bioelectrical signals that reflect muscle strength in different movement patterns. These signals are extremely weak and highly sensitive to noise, making them susceptible to various types of interference during acquisition. Therefore, analyzing and distinguishing noise sources in sEMG signals is crucial for selecting appropriate preprocessing methods to improve the signal-to-noise ratio. The main sources of noise in sEMG signals include the following:

1. Baseline Drift and Motion Artifacts [21].

When collecting sEMG signals, electrodes must be fixed near the target muscle. During limb movement, changes in muscle state cause surface fluctuations in the arm, leading to relative movement between the muscle, skin, and electrodes. This relative movement can create motion artifacts in the electromyographic signals and result in baseline drift [22].

Factors such as cable jitter, loose connections, electrode displacement due to sensor looseness, high skin impedance due to poor skin cleanliness, and reduced electrode conductivity from sweat can all contribute to baseline drift or motion artifacts. This issue is more pronounced with metal dry electrodes due to their tendency to slide relative to the skin.

2. System Noise.

Electronic components in signal acquisition devices inherently produce noise. This noise is related to the performance and manufacturing process of the components, as well as environmental factors such as temperature and humidity, and the usage duration of the components. System noise, which is random and inevitable, stems from the inherent performance of electronic components.

To remove outliers, a threshold method was employed to filter out and retain only the valid data.

The sEMG is a microelectrical signal with an amplitude in the μV range, characterized by non-linearity and non-stationarity. The signal primarily falls within the frequency band below 500 Hz, with major frequency components concentrated between 10 and 300 Hz and core energy around 20–150 Hz [23]. Due to these characteristics, sEMG signals are highly susceptible to noise interference, which significantly impacts subsequent signal processing and gesture recognition. Additionally, during signal acquisition, 50 Hz power line interference can introduce 50 Hz noise.

This experiment used a Butterworth filter for signal processing. First, a bandpass filter with a range of 10–200 Hz was applied to filter out high-frequency and low-frequency noise. Then, a 50 Hz notch filter was used to remove power line interference.

#### 2.2.2. Wavelet Denoising

Wavelet denoising is a signal processing method based on wavelet transform, known for its excellent time-frequency localization properties and suitability for handling non-stationary signals. After wavelet decomposition, the wavelet coefficient magnitudes of the signal are generally larger than those of the noise. Thus, coefficients with larger magnitudes are typically signal-dominant, while those with smaller magnitudes are largely noise [24]. Therefore, wavelet transform effectively decorrelates the data by concentrating the signal’s energy in a few large wavelet coefficients, whereas the noise energy is distributed throughout the wavelet domain. By applying a thresholding method, most noise coefficients are reduced to zero, and coefficients below the threshold are considered interference noise and removed, followed by wavelet reconstruction to achieve denoising.

#### 2.2.3. Removing Motion Artifacts

Baseline drift in sEMG signals, as explained in noise analysis, generally manifests as a very-low-frequency curve superimposed on the original signal, causing slow and slight fluctuations. This baseline drift can distort FFT analysis, correlation analysis, and power spectral density analysis, leading to spikes in low frequencies and even obscuring the main frequency components, thus affecting accuracy [12].

Collecting the baseline drift in sEMG, the least squares method can be used to minimize the sum of squared errors and find the best function fit. This trend component, representing baseline drift or other signal trends, can then be fitted and subtracted from the original signal, resulting in a new signal with baseline drift removed, thereby improving signal processing accuracy and effectiveness.

**Figure 2:**
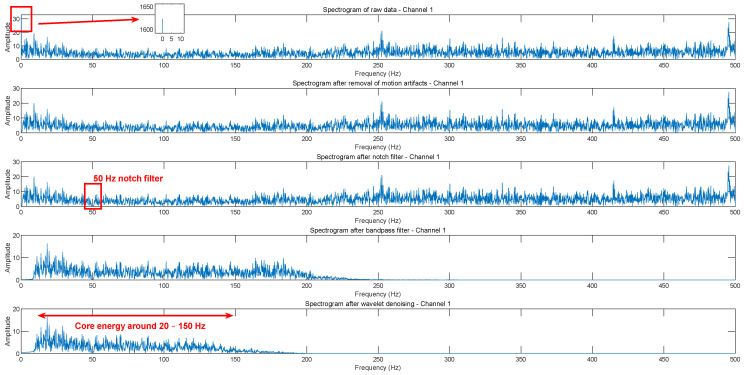
EMG Data Processing Diagram.

The results are presented in Figure 2.

### 2.3. Data Fitting and Regression

#### 2.3.1. Neural Network Time Series Fitting

Neural networks, also known as artificial neural networks (ANNs), consist of interconnected nodes or artificial neurons arranged in layers, transmitting and processing data through weighted connections.

Neural network time series fitting is a method that uses neural network models to predict or analyze time series data. The core idea is to incorporate the time dimension of the data into the neural network, allowing it to model directly over time.

The Nonlinear Auto Regressive with eXogenous inputs (NARX) neural network was applied in this study, predicting future values of time series muscle force signals (response signal F(*t*)) based on past values of both the response signal and another time series of electromyographic signals (predictive signal E(*t*)). The NARX neural network is a specific type of recurrent neural network designed for time series data, learning the time dependencies between inputs and outputs to predict future output values [25]. In the study by Serikov et al. [26], they successfully utilized the NARX network to predict nonlinear network traffic data based on time series. By employing MSE and regression R as evaluation metrics, they demonstrated the feasibility and effectiveness of their approach.

The NARX neural network consists of an input layer, hidden layers, and an output layer, utilizing the backpropagation algorithm for training and continuously optimizing the network’s weights and parameters by minimizing prediction error.

The defining equation for the NARX neural network is as follows (*Du* and *Dy* are the same, called Delay):

*y*(*t*) *= f*(*u*(*t*), *u*(*t* − 1), *u*(*t* − 2), …, *u*(*t* − *Du*), *y*(*t* − 1), *y*(*t* − 2), …, *y*(*t* − *Dy*)) (1)

In this equation, *y*(*t*) and *u*(*t*) represent the output and input of the network at time *t*, respectively. *Du* is the maximum order of input delays, and *Dy* is the maximum order of output delays. Therefore, *u*(*t* − *Du*), …, *u*(*t* − 1) are the historical inputs relative to time *t*, and *y*(*t* − *Dy*), …, *y*(*t* − 1) are the historical outputs relative to time *t*. The function *f* is the nonlinear function fitted by the network.

**Figure 3:**
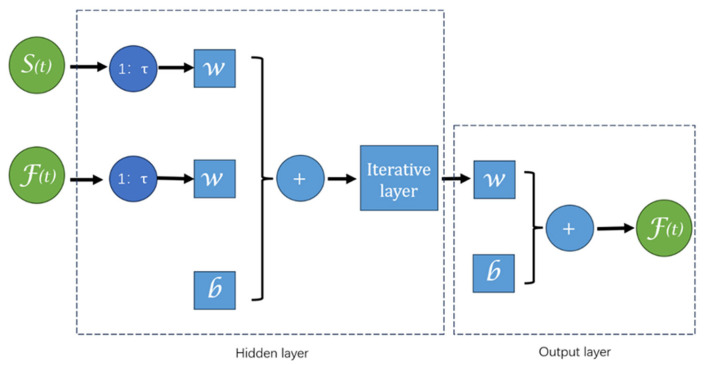
NARX neural network structure diagram.

The structure diagram of the NARX model is shown in Figure 3. The *S*(*t*) and *F*(*t*) represent the sEMG and force signals, respectively. The time delays in the NARX neural network refer to the time lag and feedback mechanism incorporated into the network. This represents the time period from the generation of the input signal to its effect on the output. This time difference can impact the network’s prediction accuracy and the system’s stability. Properly setting delay parameters helps the network better capture long-term dependencies and periodicity in time series data.

#### 2.3.2. Comparison of Different Training Methods

In this experiment, three different training strategies were compared using the same training and test sets. The parameters included a 50-layer neural network with a 1:20 time delay. The strategies were Bayesian regularized stochastic gradient descent (BRSGD), Levenberg–Marquardt (LM), and conjugate gradient (CG).

BRSGD is based on stochastic gradient descent and uses extra techniques to regularize the network towards reducing bias and variance. BRSGD introduces Bayesian regularization into the training process by adding an additional term into the loss function. This additional term penalizes the presence of large weights, which is introduced to provide a smoother network response. This method has been applied to improve the robustness and generalization performance of neural networks, especially in scenarios with limited training data. Bayesian inference naturally incorporates regularization, which aids in preventing overfitting, making it particularly advantageous in artificial neural networks [27].

The Levenberg–Marquardt (LM) algorithm is the most widely used optimization algorithm. It outperforms simple gradient descent and other conjugate gradient methods in a wide variety of problems. The algorithm is shown to be a blend of vanilla gradient descent and Gauss–Newton iteration [28].

And the CG method is a technique that lies between the steepest descent method and Newton’s method. It only requires first-order derivative information but overcomes the slow convergence of steepest descent while avoiding the need for storing, computing, and inverting the Hessian matrix as in Newton’s method. The conjugate gradient method is not only one of the most useful methods for solving large linear systems but also one of the most effective algorithms for large-scale nonlinear optimization [29].

We used the same dataset and conducted model training with two methods under identical hardware conditions (GPU NVIDIA RTX 3070, Santa Clara, CA, USA with 8 GB memory, processor AMD Ryzen 7 5800 H with Radeon Graphics at 3.20 GHz). We evaluated the two training methods based on mean squared error (MSE), regression value (R-value), and the time required for 1000 training epochs or required to reach the minimum gradient. The comparison results are shown in Table 1.

From the results, it is clear that LM demonstrated outstanding performance in model training, as evidenced by its extremely low error and a regression value of 1.0000. However, this method showed significant overfitting on the test set, making it unsuitable for practical use. As for BRSGD and CG, these two methods produced similar outcomes. While BRSGD achieved lower error and higher accuracy on the training set, the differences between the two on the test set were minimal. Notably, CG requires only 5 s for training, which is a distinct advantage.

## 3. Result

Based on the comparison of training methods described above, this experiment used the CG to analyze the impact of neural network parameters on regression results.

Overfitting and underfitting are two extreme scenarios that occur during the learning process. The impacts of underfitting include inadequate predictive power, an inability to effectively learn data features, poor generalization, and wasted potential of training data. On the other hand, overfitting occurs when a model performs well on the training data but poorly on test data or real-world data. It leads the model to learn the noise and details in the data rather than the true underlying patterns, thereby reducing its ability to generalize. Factors contributing to these issues include limited or homogeneous training samples, excessive noise in the data, and overly complex models [30,31]. Similarly, excessive noise in the training data can distract the model from the true input–output relationships, and a model that is too complex may memorize the training data but fail to generalize to unseen data [32]. Considering the goal of reducing training costs and shortening training time, methods that require huge resources for repeated training of the model are not ideal. In addition, compared to the misjudgments caused by overfitting, accuracy fluctuations due to underfitting are relatively more acceptable. Therefore, we balanced the number of network layers, ensuring that the network depth did not exceed 30 layers.

The following data (Table 2) present the test results under different network parameters. Following the study by Serikov et al. [26], we also adopted MSE and regression R as the primary evaluation metrics, while adding MAE as an additional metric.

For different model layers, the average performance of time delay was calculated, and the results show that the shallow model had better performance and the deep model had bad performance. This means that overfitting may have occurred during layer increasing.

**Figure 4:**
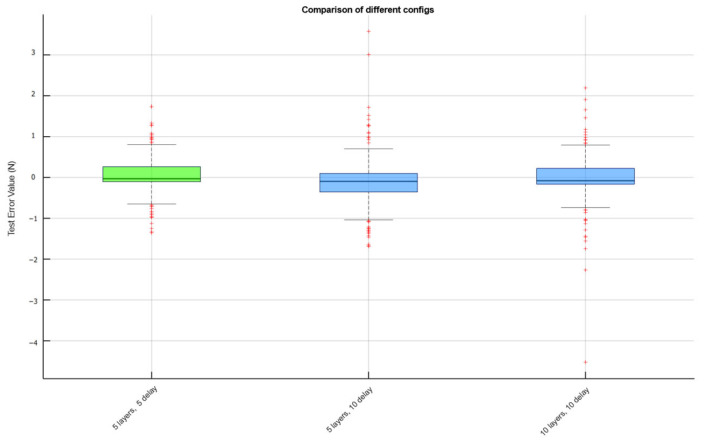
The comparison chart of EMG after processing through each step (The red “+” was out lier).

Three parameter combinations showed excellent test results, namely, (5 layers, 5 delay), (5 layers, 10 delay), and (10 layers, 10 delay). The error distribution of these three results is displayed using box plots as Figure 4.

**Figure 5:**
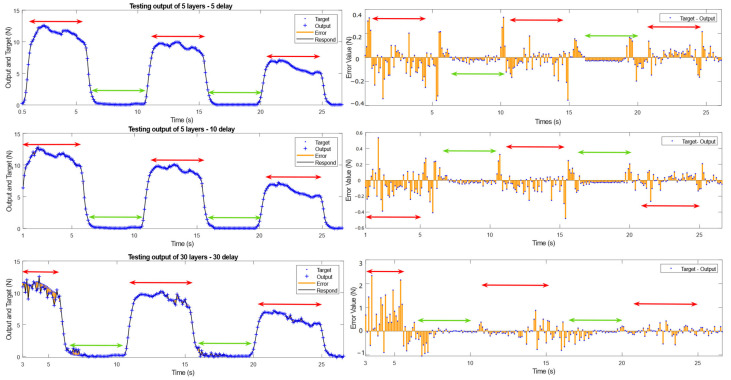
Testing results as an example (red arrows indicating “Grip Hold” and green arrows indicating “Grip Release”).

This paper demonstrates the fitting result using examples of (5 layers, 5 delay), (5 layers, 10 delay), and (30 layers, 30 delay) as follows Figure 5.

Under the (5 layers, 5 delay) testing conditions, it was observed that the initial fitting results were not ideal, but they improved over time. This is because, at the beginning, the number of sEMG signal samples available for analysis was limited, making accurate results difficult. As time progressed, the number of usable samples stabilized, leading to more accurate fitting results. The model shows sensitivity to the nonlinear dynamic changes in force, accurately describing the dynamic variations even during the grip maintenance phase.

Additionally, the model is also relatively sensitive to the start of force application and release. It fits the linear changes in force very precisely, accurately capturing the duration of force application and release.

Under the conditions of (5 layers, 10 delay), it can be observed that the initial fitting error was further amplified. The larger fitting errors were concentrated at the start and end points of the grip hold and grip release phases. However, the model still performed well in fitting the linear changes in grip strength and the fluctuations in grip strength during the maintenance state.

Under the (30 layers, 30 delay) testing conditions, the errors observed in the previous group were significantly amplified. Additionally, there was a noticeable deviation in fitting the linear changes in grip strength, making it difficult to accurately estimate the rate of grip strength changes during force application and release. Moreover, during the third force application period, the model showed a decreased sensitivity to irregular fluctuations in grip strength during the maintenance phase, leading to less accurate estimations.

## 4. Discussion

The results indicate that the BP algorithm and time-series-based neural network fitting effectively predicted grip strength from sEMG signals. The model performed well in both the linear changes during force application and release, as well as in grip maintenance phases, especially under the (5 layers, 5 delay) conditions, with minimal overall error and high sensitivity to nonlinear fluctuations. The model’s generalizability was supported by its consistent performance across different subjects. The best performance was MSE = 0.17, MAE = 0.72, and R = 0.9991 when the model had 5 layers and 5 delay.

However, there are notable limitations. Initially, there was a significant deviation between the predicted and actual results in the first few data points. This issue was likely due to the baseline grip strength estimation being based on data points approximately 10 samples before the first force application, leading to insufficient samples for accurate grip strength estimation during the maintenance phase with the delay-involved neural network analysis. The initial prediction is highly dependent on the start-up setting. So, this can be improved by an adaptation strategy and will be done in the future.

Additionally, significant deviations were observed in the latter part of the grip maintenance phase. In this experiment, subjects were instructed to maintain maximum grip on the sensor for 5 s. Sustaining maximum grip imposes a considerable burden on the muscles, leading to potential interference in the sEMG signals during the latter part of the maintenance phase, which affects the model’s prediction accuracy.

One possible factor is that the sEMG signal output mechanisms during force application and prolonged maintenance may differ. Specifically, the signal logic might change in the last few seconds of the 5 s period, and since subjects quickly transitioned to the release phase after 5 s, there were fewer samples available for analyzing the prolonged maintenance phase. These limited data may hinder the model’s ability to accurately interpret these signals. Another potential factor is the special changes in sEMG signals under muscle fatigue [33]. The literature suggests that fatigued muscles exhibit different sEMG signal patterns compared to non-fatigued muscles, likely due to interference from peripheral nervous system signals aimed at preventing excessive muscle fatigue. With a limited sample size, the model may struggle to adequately analyze these fatigue-related signal variations.

A third significant deviation occurred during the initial part of the force release phase. In this experiment, grip strength was measured using a traditional method where subjects grip a sensor. Given practical conditions, it is possible that subjects might have removed their fingers from the sensor before completing the release phase (despite instructions to keep their fingers on the sensor throughout the test). As a result, even though the sensor detects zero force, the subject’s actual grip strength has not yet reached zero, and hand movements may not have fully ceased. Consequently, the sEMG signals at this point do not stabilize.

In such non-ideal scenarios, the model may interpret these sEMG signals as indicating that the subject is still exerting force. From a realistic simulation perspective, this outcome could be considered more accurate and reflective of the actual situation. However, from an idealized experimental perspective, where the sensor should have recorded zero force, this discrepancy creates a conflict between the sensor data and the model’s predictions.

The experimental results indicate that increasing the number of neural network layers did not positively impact the fitting prediction outcomes. It is suspected that this increase led to overfitting, as evidenced by a noticeable drop in the regression R-value, reducing the model’s sensitivity to irregular fluctuations in grip strength during the maintenance phase. Additionally, excessively long-time delays also decreased the accuracy of the predictions. According to the study of Beck et al., only sEMG signals from a specific previous time period affect current muscle strength changes [34]. Thus, a time delay closer to the length of this period yields more accurate results. Conversely, too short a time delay leads to insufficient sample size for analysis, while too long a delay results in redundant information, complicating the assessment of current muscle strength. Thus, for this experiment, 10 layer and 20 time delay are the best parameters.

LSTMs and GRUs are widely used deep learning models for time-series data. LSTMs [35] is an enhanced version of RNN, specifically designed to overcome the vanishing and exploding gradient issues that arise when processing long sequences. By incorporating memory cells and multiple gating mechanisms, LSTMs effectively capture long-term dependencies, making it highly suitable for tasks like machine translation, text generation, and speech recognition.

GRUs [36], in contrast, are a simplified variant of LSTMs. They eliminate some of the complexity while retaining essential gating mechanisms, enabling long-term dependencies to be handled efficiently. GRUs’ strengths lie in their simpler architecture, fewer parameters, and faster training speed, with performance comparable to, or even better than, LSTMs in certain tasks. Their areas of application largely overlap with those of LSTMs.

In this exploratory study, we chose the NARX network model for its unique advantages. NARX networks excel at modeling the relationship between inputs and outputs intuitively, offering strong interpretability. With fewer parameters, they are easier to train and less prone to overfitting, even with small datasets, making them particularly well-suited to our limited sample size. The dataset used in this study allowed the model to perform well. However, the limited size of the training data constrained the model’s complexity, increasing the risk of overfitting. This limits the model’s ability to generalize, as it may fit well to training data but struggle with unseen test data [37]. To address this, future work will focus on collecting more data to expand the dataset and improve the model’s generalization capabilities, reducing overfitting and enhancing overall performance.

That said, the NARX network has inherent limitations. It requires manual tuning of the time delay parameter, which involves considerable trial and error to determine the optimal setting. Furthermore, NARX struggles with very-long-term dependencies due to its lack of dynamic memory capabilities, making it less adaptable to data with complex temporal correlations.

In practical applications, long-term device use by participants often involves handling extended dependencies and addressing issues such as muscle fatigue from sustained effort. The NARX network may fall short in managing such long-term relationships. Additionally, this study did not collect data on prolonged muscle exertion, and the predictions made here do not currently address muscle fatigue explicitly. Future research could explore the use of GRUs for modeling extended muscle fatigue states using EMG data. GRUs’ suitability for processing large-scale, long-term data makes it a promising candidate for targeted experiments and investigations in this domain.

To achieve more accurate prediction results (e.g., avoiding EMG variations caused by muscle fatigue), future potential improvements include developing a multimodal model that integrates EEG data. Many studies have shown that EEG signals also play an important role in controlling local muscles [38,39]. Therefore, in future work, we will consider building a multimodal large model that uses EEG signals and EMG signals as two heterogeneous data inputs to obtain better inference results.

Prediction of dynamic grip strength based on sEMG has notable potential for practical applications. For example, in the realm of smart prosthetics, it could enhance the ability to simulate real human grip strength, thus improving the precision of tasks requiring accurate grip control. In virtual reality, it can accurately capture and reflect human posture and force, leading to more effective interactions with virtual environments and better physical simulations within these virtual settings.

Bardizbanian et al. [12] suggested that fewer than 16 electrodes could still yield ideal prediction results. Our study has confirmed this, showing that with just six electrodes and three channels, we can achieve over 95% accuracy. Additionally, compared to their training time of nearly 1 min, we successfully reduced the training time to under 5 s. Given that current smart prosthetic devices typically use 12–16 electrodes [40], we believe the findings of this study have significant value for the development of lightweight prosthetic devices. The short training and response times could provide an enhanced user experience in real-world applications.

In the field of virtual reality, Zhang et al. [41] collected eight-channel EMG data from the fingertips and analyzed force actions primarily involving the fingers (e.g., pressing, pinching). Their research achieved about 90% accuracy in predicting finger strength. To address the generalization issue of large data models when applied to new users, they used a transfer learning strategy, achieving an average accuracy of over 85%. In contrast, our study effectively solved the force prediction for palm-dominant actions (e.g., gripping), and thanks to the advantages of small datasets and short training times, it allows for a one-time model construction for each user, ensuring a tailored and seamless human-machine interaction experience.

## 5. Conclusions

This study successfully applied the time series-based NARX network to fit and predict the nonlinear dynamic changes in upper limb grip strength based on sEMG signals. This represents a small step forward in exploring the decoding of force from sEMG signals, though there remains considerable room for improvement and further research.

To address the initial instability in prediction results, a lead time could be incorporated in practical applications, allowing the model to adapt to the user’s sEMG signals before fully engaging in prediction tasks. For addressing prediction deviations caused by prolonged grip strength maintenance, further investigation into muscle fatigue phenomena is warranted. Collecting training data with extended grip maintenance times could provide more sEMG signals and grip strength information under fatigue conditions, aiding the model in analyzing the mapping between sEMG signals and muscle strength in such environments.

To correct prediction deviations during the initial part of the release phase, an output detection threshold could be established. Minor grip strength fluctuations that do not exceed this threshold could be classified as zero, thereby addressing the issue of the model predicting small grip strength values even after force release is completed.

This technology holds promising application prospects for nonlinear dynamic grip strength prediction. It has the potential to significantly impact the development and enhancement of smart prosthetics, as well as the simulation of physical scenarios and optimization of human–computer interactions in virtual reality settings.

## Figures and Tables

**Table 1:** Comparison of BRSGD, LM, and CG.

Training Method	MSE (N^2^)		RMSE (N)_		MAE (N)		R-Value		Training Time
	Training Set	Testing Set	Training Set	Testing Set	Training Set	Testing Set	Training Set	Testing Set	
BRSGD	0.0045	0.2335	0.0670	0.4831	0.0328	0.2448	0.9999	0.9941	45 min 25 s
LM	6.616 × 10^−18^	85.3096	2.572 × 10^−9^	9.2363	4.827 × 10^−10^	5.1816	1.0000	0.3510	9 min 30 s
CG	0.0162	0.2181	0.1272	0.4670	0.0842	0.2330	0.9996	0.9944	5 s

**Table 2:** MSE, MAE and regression value under different parameters.

Delay	Number of Neural Network Layers											
	5			10			20			30		
	MSE (N^2^)	MAE (N)	R	MSE (N^2^)	MAE (N)	R	MSE (N^2^)	MAE (N)	R	MSE (N^2^)	MAE (N)	R
**5**	**0.17**	**0.72**	0.9991	2.42	1.62	0.9940	3.09	1.83	0.9924	13.1	2.40	0.9673
10	0.28	0.87	**0.9994**	0.82	1.37	0.9980	1.60	1.86	0.9960	1.72	1.87	0.9957
20	0.87	1.36	0.9977	1.13	1.72	0.9970	0.96	1.79	0.9977	0.87	1.52	0.9980
30	0.34	1.34	0.9992	0.93	1.93	0.9978	1.70	2.20	0.9952	2.80	2.42	0.9925
40	1.23	1.97	0.9966	0.93	1.79	0.9971	1.47	2.03	0.9958	4.50	2.74	0.9886
Average value	0.58	1.25	0.9985	1.25	1.69	0.9968	1.76	1.94	0.9954	5.10	2.19	0.9884

MSE (×10^−1^); MAE (×10^−1^); highlight (bold): the best results for each metric.

## Data Availability

The data presented in this study is available on request from the corresponding author.

## References

[1] Chowdhury R.H., Reaz M.B.I., Ali M.A.B.M., Bakar A.A.A., Chellappan K., Chang T.G. (2013). Surface Electromyography Signal Processing and Classification Techniques. Sensors.

[2] Young A.J., Gannon H., Ferris D.P. (2017). A biomechanical comparison of proportional electromyography control to biological torque control using a powered hip exoskeleton. Front. Bioeng. Biotechnol..

[3] Ma R., Zhang L., Li G., Jiang D., Xu S., Chen D. (2020). Grasping force prediction based on sEMG signals. Alex. Eng. J..

[4] McCool P., Petropoulakis L., Soraghan J.J., Chatlani N. (2015). Improved pattern recognition classification accuracy for surface myoelectric signals using spectral enhancement. Biomed. Signal Process. Control.

[5] NN Unanyan A.A. (2021). Belov. Design of upper limb prosthesis using real-time motion detection method based on EMG signal processing. Biomed. Signal Process. Control.

[6] Kuiken T.A., Li G., Lock B.A., Lipschutz R.D., Miller L.A., Stubblefield K.A., Englehart K.B. (2009). Targeted Muscle Reinnervation for Real-time Myoelectric Control of Multifunctional Artificial Arms. J. Am. Med. Assoc. (JAMA).

[7] Wang S., Zheng J., Zheng B., Jiang X. (2022). Phase-Based Grasp Classification for Prosthetic Hand Control Using sEMG. Biosensors.

[8] Kim S., Chung W.K., Kim K. SEMG-based static force estimation for human-robot interaction using deep learning. Proceedings of the 2020 17th International Conference on Ubiquitous Robots (UR).

[9] Wu Y., Liang S., Yan T., Ao J., Zhou Z., Li X. (2022). Classification and simulation of process of linear change for grip force at different grip speeds by using supervised learning based on sEMG. Expert Syst. Appl..

[10] Jiang D., Li G., Sun Y., Kong J., Tao B. (2019). Gesture recognition based on skeletonization algorithm and CNN with ASL database. Multimed. Tools Appl..

[11] Scheme E., Englehart K. (2011). Electromyogram dynamical characteristics of sEMG signals of hand grasps via recurrence plot pattern recognition for control of powered upper-limb prostheses: State of the art and challenges for clinical use. J. Rehabil. Res. Dev..

[12] Bardizbanian B., Zhu Z., Li J., Huang X., Dai C., Martinez-Luna C., McDonald B.E., Farrell T.R., Clancy E.A. Efficiently training two-DoF hand-wrist EMG-force models. Proceedings of the 2020 42nd Annual International Conference of the IEEE Engineering in Medicine & Biology Society (EMBC).

[13] Sharma T., Sharma K.P., Sharma T. (2024). Identification and Classification of Myoelectric Signal Features Related to Hand Motions. Neurophysiology.

[14] Huang R., Xue X., Xiao R., Bu F. (2023). A novel method for ecg signal compression and reconstruction: Down-sampling operation and signal-referenced network. Electronics.

[15] Lieber R.L., Jacobson M.D., Fazeli B.M., Abrams R.A., Botte M.J. (1992). Architecture of selected muscles of the arm and forearm: Anatomy and implications for tendon transfer. J. Hand Surg..

[16] Palastanga N., Field D., Soames R. (2006). Anatomy and Human Movement: Structure and Function.

[17] Schieber M.H. (1995). Muscular production of individuated finger movements: The roles of extrinsic finger muscles. J. Neurosci..

[18] Ma N., Kumar D.K., Pah N. Classification of hand direction using multi-channel electromyography by neural network. Proceedings of the Seventh Australian and New Zealand Intelligent Information Systems Conference.

[19] Kuran B. (2019). Functional assessment in hand with flexor and extensor tendon injuries. Hand Function: A Practical Guide to Assessment.

[20] Peleg D., Braiman E., Yom-Tov E., Inbar G.F. (2002). Classification of finger activation for use in a robotic prosthesis arm. IEEE Trans. Neural Syst. Rehabil. Eng..

[21] Huang R., Woo S.-W., Hong K.-S. Real-time Motion Artifacts and Low-Frequency Drift Correction for Functional Near-infrared Spectroscopy. Proceedings of the 2020 12th International Conference on Computer and Automation Engineering (ICCAE 2020), Association for Computing Machinery.

[22] Chu J.U., Moon I., Lee Y.J., Kim S.K., Mun M.S. (2007). A supervised feature-projection-based real-time EMG pattern recognition for multifunction myoelectric hand control. IEEE-ASME Trans. Mechatron..

[23] Vijayvargiya A., Singh B., Kumar R., Tavares J.M.R. (2022). Human lower limb activity recognition techniques, databases, challenges and its applications using sEMG signal: An overview. Biomed. Eng. Lett..

[24] Wang J., Yang L., Gao L., Miao Q. Current progress on weak signal detection. Proceedings of the 2013 International Conference on Quality, Reliability, Risk, Maintenance, and Safety Engineering (QR2MSE).

[25] Kadochnikova A., Zhu Y., Lang Z.Q., Kadirkamanathan V. (2023). Integrated Identification of the Nonlinear Autoregressive Models with Exogenous Inputs (NARX) for Engineering Systems Design. IEEE Transactions on Control Systems Technology.

[26] Serikov T., Zhetpisbayeva A., Mirzakulova S., Zhetpisbayev K., Ibrayeva Z., Tolegenova A., Soboleva L., Zhumazhanov B. (2021). Application of the NARX neural network for predicting a one-dimensional time series. East. Eur. J. Enterp. Technol..

[27] Truong T.T., Airao J., Hojati F., Ilvig C.F., Azarhoushang B., Karras P., Aghababaei R. (2024). Data-driven prediction of tool wear using Bayesian regularized artificial neural networks. Measurement.

[28] Ranganathan A. (2004). The levenberg-marquardt algorithm. Tutoral LM Algorithm.

[29] Nazareth J.L. (2009). Conjugate gradient method. WIREs Comput. Stat..

[30] Li H., Li J., Guan X., Liang B. Research on overfitting of deep learning. Proceedings of the 2019 15th International Conference on Computational Intelligence and Security (CIS).

[31] Ghasemian A., Hosseinmardi H., Clauset A. (2019). Evaluating overfit and underfit in models of network community structure. IEEE Trans. Knowl. Data Eng..

[32] Aliferis C., Simon G. (2024). Overfitting, Underfitting and General Model Overconfidence and Under-Performance Pitfalls and Best Practices in Machine Learning and AI. Artificial Intelligence and Machine Learning in Health Care and Medical Sciences: Best Practices and Pitfalls.

[33] Suganthi J.R., Rajeswari K. Evaluation of Muscle Fatigue based on SEMG using Deep Learning Techniques. Proceedings of the 2023 5th International Conference on Inventive Research in Computing Applications (ICIRCA).

[34] Beck T.W., Stock M.S., DeFreitas J.M. (2012). Time-Frequency Analysis of Surface Electromyographic Signals During Fatiguing Isokinetic Muscle Actions. J. Strength Cond. Res..

[35] Hochreiter S. (1997). Long Short-Term Memory.

[36] Cho K., Van Merriënboer B., Gulcehre C., Bahdanau D., Bougares F., Schwenk H., Bengio Y. (2014). Learning phrase representations using RNN encoder-decoder for statistical machine translation. arXiv.

[37] Ying X. (2019). An overview of overfitting and its solutions. J. Phys. Conf. Ser..

[38] Rizzo R., Garcia-Retortillo S., Ivanov P.C. (2022). Dynamic networks of physiologic interactions of brain waves and rhythms in muscle activity. Hum. Mov. Sci..

[39] Penn A.A., Shatz C.J. (1999). Brain waves and brain wiring: The role of endogenous and sensory-driven neural activity in development. Pediatr. Res..

[40] Parajuli N., Sreenivasan N., Bifulco P., Cesarelli M., Savino S., Niola V., Esposito D., Hamilton T.J., Naik G.R., Gunawardana U. (2019). Real-time EMG based pattern recognition control for hand prostheses: A review on existing methods, challenges and future implementation. Sensors.

[41] Zhang Y., Liang B., Chen B., Torrens P.M., Atashzar S.F., Lin D., Sun Q. (2022). Force-aware interface via electromyography for natural VR/AR interaction. ACM Trans. Graph. (TOG).

